# The Cell Research Trends of Asthma: A Stem Frequency Analysis of the Literature

**DOI:** 10.1155/2018/9363820

**Published:** 2018-08-23

**Authors:** Wenchao Tang, Yi Shang, Bin Xiao, Peitong Wen, Ruoyun Lyu, Ke Ning

**Affiliations:** ^1^School of Acupuncture-Moxibustion and Tuina, Shanghai University of Traditional Chinese Medicine, Shanghai 201203, China; ^2^Department of Electrical Engineering and Computer Science, University of Missouri, Columbia, MO 65211, USA; ^3^School of Basic Medical Sciences, Shanghai University of Traditional Chinese Medicine, Shanghai 201203, China

## Abstract

**Objective:**

This study summarized asthma literature indexed in the Medical Literature Analysis and Retrieval System Online (MEDLINE) and explored the history and present trends of asthma cell research by stem frequency ranking to forecast the prospect of future work.

**Methods:**

Literature was obtained from MEDLINE for the past 30 years and divided into three groups by decade as the retrieval time. The frequency of stemmed words in each group was calculated using Python with Apache Spark and the Natural Language Tool Kit for ranking. The unique stems or shared stems of 3 decades were summarized.

**Results:**

A total of 1331, 4393, and 7215 records were retrieved from 3 decades chronologically, and the stem ranking of the top 50 were listed by frequency. The number of stems shared with 3 decades was 26 and with the first and last 2 decades was 5 and 13.

**Conclusions:**

The number of cell research studies of asthma has increased rapidly, and scholars have paid more attentions on experimental research, especially on mechanistic research. Eosinophils, mast cells, and T cells are the hot spots of immunocyte research, while epithelia and smooth muscle cells are the hot spots of structural cell research. The research trend is closely linked with the development of experimental technology, including animal models. Early studies featured basic research, but immunity research has dominated in recent decades. The distinct definition of asthma phenotypes associated with genetic characteristics, immunity research, and the introduction of new cells will be the hot spots in future work.

## 1. Introduction

Asthma is a major public health problem around the world, affecting individuals across the age spectrum from infants to older adults. Therefore, research on its pathogenesis and treatment has been a hot topic in the study of respiratory diseases. It is now well accepted that cell activity has a close relationship with pathogenesis of asthma, and numerous basic and clinical studies focus on different types of relevant cells.

As a typical example of type I hypersensitivity, the research of immune cells concerned with asthma, such as lymphocytes, monocytes, and mast cells, is most common. For instance, T cell subsets include CD4⁺, CD8⁺ T cells [1], Th2, Th17 [2], Th9 [3], and so on. Except for immune-relevant cells, structural cells of the airways, such as epithelial cells [4], smooth muscle cells [5], and bronchial myofibroblasts [6], are also an important focus of research. In recent years, cell therapy has attracted the attention of researchers to treat asthma and its complications. A study revealed that bone marrow-derived mesenchymal stem cell (BMSC) therapy significantly suppressed lung pathology and inflammation in the ovalbumin-induced asthma mouse model [7].

Currently, there has been continued interest in targeting airway cells for developing new asthma treatments. Therefore, it has become imperative to analyze the current trend and future direction of asthma cell research. This study summarized asthma literature indexed in the Medical Literature Analysis and Retrieval System Online (MEDLINE) of the National Library of Medicine (NLM) in the past 30 years and explored the history and present state of asthma cell research by stem frequency rank to provide ideas for future work.

## 2. Objects and Methods

### 2.1. Objects

Literature of asthma cell research indexed from MEDLINE in the past 30 years was divided into three groups with 10 years as the retrieval time. The literature containing the keywords “Asthma” and “Cell” in the fields “Title” or “Abstract” was included for further investigation. The limit of publication date in the three groups were “January 1, 1987, to December 31, 1996,” “January 1, 1997, to December 13, 2006,” and “January 1, 2007, to December 31, 2016,” respectively.

The search results of each decade were exported into a CSV file with information such as title and author. All the titles of each CSV file were saved as a text file for analysis with stem frequency rank.

### 2.2. Programming

Due to the large amount data in the literature, we adopted Apache Hadoop, which is commonly used in big data analysis as the data storage framework. As a file system supporting a data-intensive distributed application, Apache Hadoop has better distribution characteristics and provides file services with both reliability and mobility for the program development [8]. To speed up the computation, we selected the Apache Spark open-source computing framework in our study instead of the Apache Hadoop built-in MapReduce computing method. The major difference between Spark and MapReduce lies in in-memory computing technology, which means the data are analyzed and processed to acquire the results in the memory before being written to the hard disk [9].

The analysis of stem frequency ranking was handled using the Natural Language Tool Kit (NLTK). NLTK is an important tool for dealing with human natural language, which can be applied to word merging, text retrieval, and statistics, and so on. The technologies such as “Word frequency Accumulation,” “Stemming Processing,” and “Stop-word Filtering” applied in this study were all performed with NLTK [10]. According to the integrated application of the above techniques, the programming environment and working process can be summarized as follows:

1. Programming environment: EC2 server of Amazon Web Services (AWS) platform was selected as the programming environment.
    Server model: t2 micro
    Server Location: Oregon, United States
    Operating System and software: Ubuntu Server 14.04 with built-in Python language (version 2.7.3), Apache Spark (version 1.6.2), and NLTK (version 3.0).
2. Working process:
  - Import a text file.
  - Create the Spark context.
  - Convert all text to lowercase.
  - Remove punctuations, empty lines, and nonletter symbols.
  - Use stop word list to filter irrelevant vocabulary of research such as “they,” “where,” “to,” and “is.” The words influencing the results such as “review,” “asthma,” and “cell” were also added into the stop word list for filtering.
  - Stemming for reducing each word to its base form by removing its common morphological ending. In this study, we utilized PorterStemmer, a Python wrapper of the libstemmer library, to perform the stemming step.
  - Rank all stems according to their frequency.
  - List the top 50 stems, and output the results.

## 3. Results

### 3.1. The Number of Studies Retrieved by Searching Three Decades

1331, 4393, and 7215 records were retrieved in the 1st, 2nd, and 3rd decade, respectively, which shows that the number of cell research literature of asthma indexed by MEDLINE presents explosive growth from 1987 to 2017; the literature number of the next decade was 1.5–2 times greater than the previous one.

### 3.2. The Top 50 Stems of Three Decades

**Figure 1:**
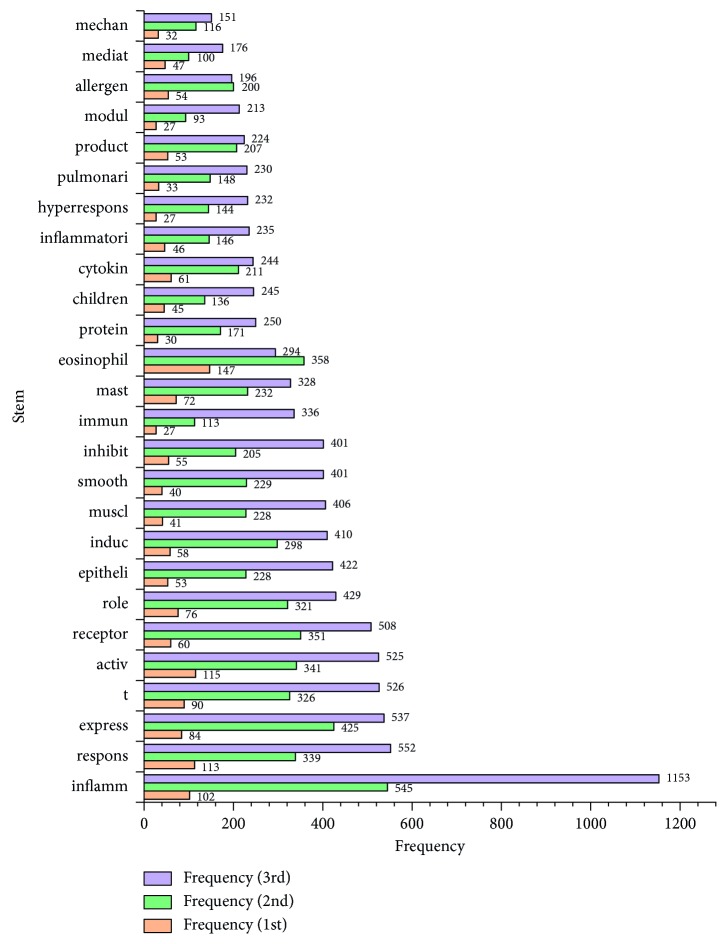
The shared stems of three decades. The orange, green, and purple bars show the frequencies of stems in the 1st, 2nd, and 3rd decade, respectively.

**Figure 2:**
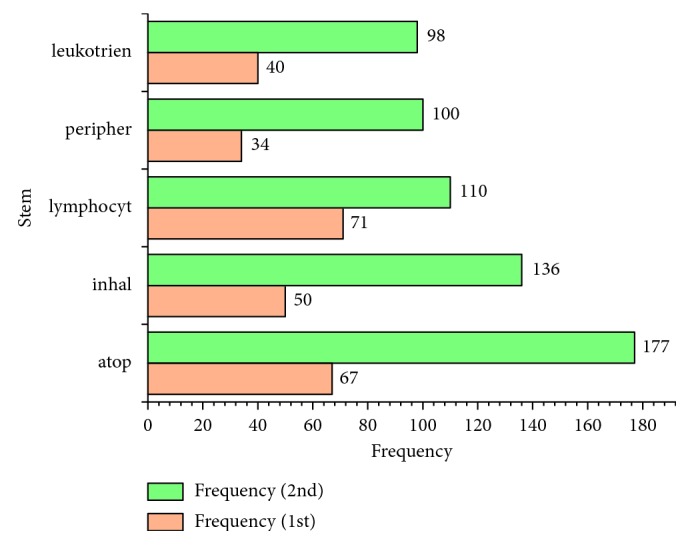
The shared stems of the first two decades. The orange and green bars show the frequencies of stems in the 1st and 2nd decade, respectively.

**Figure 3:**
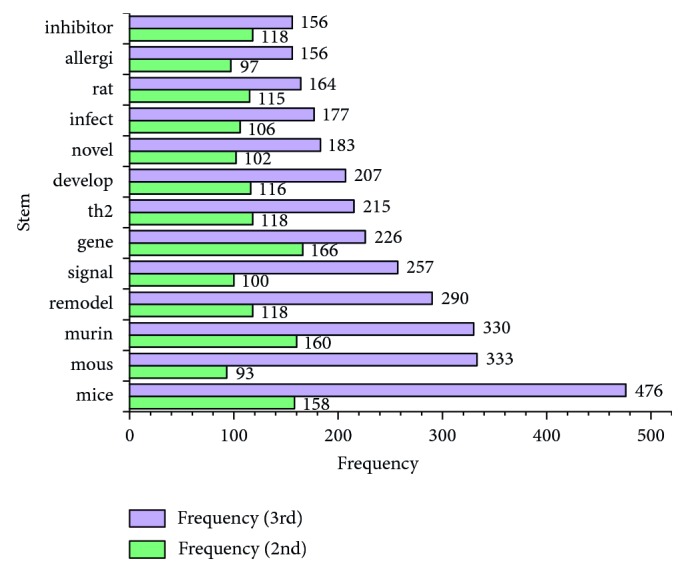
The shared stems of the last two decades. The green and purple bars show the frequencies of stems in the 2nd and 3rd decade, respectively.

**Figure 4:**
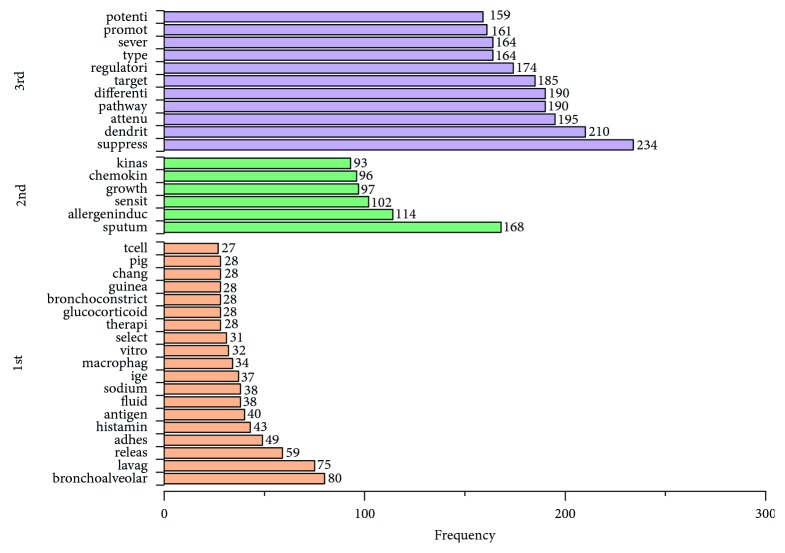
The unique stems of three decades. The orange, green, and purple bars show the frequencies of stems in the 1st, 2nd, and 3rd decade, respectively.

The top 50 stems of 3 decades are listed in Table 1. The number of stems shared with 3 decades was 26 (Figure 1) and with the first and last 2 decades were 5 and 13 (Figures 2 and 3). The numbers of unique stems of 3 decades were 19, 6, and 11, respectively (Figure 4). According to the chronological order, the author names with the highest frequency of the three decades are Pascal Chanez (Aix-Marseille University, Paris, France), Stephen T. Holgate (University of Southampton, Southampton, United Kingdom), and Andrew Halayko (University of Manitoba, Manitoba, Canada).

## 4. Discussion

### 4.1. Mainstream Research Trends in Three Decades

The mainstream research trends can be summarized from stems shared with 3 decades. First, experimental research attracted more attention by researchers rather than clinical research. “Children” is the only relevant stem on behalf of clinical research for its frequent occurrence of asthma among children, and a study reported that asthma is common in children and is a leading cause of childhood hospitalization [11]. The stems related with experimental research, such as “respons,” “express,” and “induc,” were much more common with rising frequency year after year. Mechanism (mechan) and interventional effect were the two main directions of experimental research, but mechanism was more popular among scholars because stems about activation (activ) of cells or pathways [12–14], immune or cell response (respons) [15–17], genetic or protein expression (express) [18–20], and the role of cells or relevant genes or protein were always in the top 10 among all decades. The other 3 stems about mechanism research “product,” “induc,” and “mediat” were mainly frequent in “production of cytokines” [21], “protein or allergen-induced” [22], and “cell-mediated” [23], respectively. Inhibition was a typical intervention effect and its stem “inhibit” was ranked in our results. For example, the following were included: the inhibition of glucocorticoids on degranulation of mast cells in allergic asthma [24], inhibition of the kinase ITK in a mouse model of asthma reduces cell death [25], and the inhibition of CD38 gene-modified dendritic cells on murine asthma development [26].

Second, two frequent stems about pathologic changes of asthma were “inflamm” and “hyperrespons.” “Inflamm” was also in the top 10 because airway inflammation is the main expression of asthma, with mechanism research about inflammation such as etiological agents and influence factor [27, 28], being very common. Hyperresponsiveness was often mentioned with inflammation [29, 30] for immunology-related study of asthma.

Third, in terms of different types of cells related with asthma, eosinophils, mast cells, and T cells are the hot spots of immunocytes, according to the results of ranking. Mast cells are the “first line of defense” in which innate/adaptive immune cells can be activated to release a wide range of mediators by allergen-IgE-specific triggers and are widely distributed in tissues of the airway exposed to the environment, so mast cells preempt the critical roles played by histamine and mucus secretion in causing airway obstruction [31, 32]. The studies about the expression of CD antigens [33–35] involving mast cells and its mediated cytokines [36] are very common. Airway eosinophilias are associated with the inflammatory response and likely participate in airway remodeling [37–40]. Many studies have reported that the expression of its granular proteins has functions relevant to the features of asthma, including histopathologic changes, reversible airway narrowing, and bronchial hyperreactivity [41–44]. The activated T cells in the airway wall are associated with inflammation of asthma [45, 46], and the subsets of T cell antigens have attracted extensive attention by researchers, such as the T cells of CD4+(T helper) [47–51], CD8+ [52–54], CD25+ [55], CD28 [56, 57], CD29 [58, 59], CD39+, and CD73 [60–63]. The imbalance of different subsets and the regulatory mechanism are the research emphasis of this field [64–67].

Epithelia and smooth muscle cells (SMCs) are the hot spots of structural cell studies. Research has shown that airway epithelial barrier dysfunction may have important implications for asthma [68–72]. The relevant genes or protein expression of epithelia and the regulatory mechanism of barrier function or dysfunction are the research emphasis [27, 28]. It has been reported that SMCs isolated from asthma patients release more proinflammatory mediators than that in control subjects [73], which may contribute to airway wall remodeling [74].

### 4.2. Variation Trends of Research over Time

Several variation trends can be summarized after comparing the shared stems in the first and last two decades.

First, the phenotype definition of asthma has become gradually clearer. The shared stem “atopic” in the first two decades showed that “atopic” and “non-atopic” stems were often used to define the phenotypes of asthma due to the limited available data about asthma and atopy at that time [75], which resulted in ambiguity of the phenotype definition. However, the stem “allergi” shared with the last two decades indicated that the concept “allergic asthma” was widely used in studies [76, 77], which indicates that the phenotype of asthma was definitive.

Second, genetic studies and airway remodeling have received more attention. Along with novel experiment technologies applied in molding and detection, more studies of signaling pathways [23] and airway remodeling [78] at the gene level [18] were performed in the last 2 decades with evidence from the common stems “gene,” “signal,” and “remodel.” Moreover, the shared stems “mice,” “rat,” “murine,” and “mous” in the last two decades have shown that more animal models of rats were used in such experimental research [79–82]. In contrast, the shared stems of the first two decades implied studies of downstream signaling pathways including cytokines [83], leukotrienes [84], peripheral blood [85], or different types of receptors [86] attracted great attention at that time.

Finally, looking into changes in therapeutic approaches, the shared stem “inhal” in first two decades showed that inhaled treatment was mainstream at the early stage [87, 88]. However, it ceased to be the hot spot because of the continuous exploration of new treatments or drugs, such as Montelukast [89] and monoclonal antibodies [90]. During the past 20 years, the shared stem, “inhibitor,” indicated that as one type of a new drug for asthma, inhibitors such as histone deacetylase inhibitors [91, 92] and tyrosine kinase inhibitors [93] were implicated in influencing gene expression of asthma-related cytokines [94], gaining importance.

### 4.3. Distinctive Research Hot Spots of Every Decade

Several distinctive research hot spots can be analyzed according to the unique stems of each decade. Two specific aspects were concerned in the studies of the first decade. First, the relevant mechanism researches including the release of cytokines [95] or histamines [95] and cell adhesion [96] about bronchoalveolar and macrophages [97, 98] were performed with corresponding experimental approaches such as cell counting method, immunofluorescence, ELISA, and bioassay commonly [99]. Second, there was specific phenomenon that researchers were enthusiastic about asthma therapy, and glucocorticoid [100] and nedocromil sodium [101] were often studied using pulmonary function test.

The main hot spot drawn from the unique stems of the second decade is that the allergen-induced topics, such as airway hyperresponsiveness [102] or inflammation and the regulation mechanism of allergic sensitization [103], were popular. Its relevant common experimental techniques included immunohistochemistry, flow cytometry, RT-PCR, and ELISA [104]. In addition, the specific research topics “different types of growth factors” [105], “kinases” [106], and “chemokines” [107] were also common due to their relevant roles that have been gradually explored and affirmed.

With the development of genetic technology, the research of the immune response became prevalent in the third decade, and specific stems about its mechanism, regulation, and signaling pathways such as “pathway” [108], “target” [109], and “regulatori” [110] were found in the ranking. In terms of the corresponding experimental methods, some new techniques including digital droplet PCR (ddPCR) [111], whole-genome screen [112], and multiplexed fluorescent microsphere-based immunoassay (xMAP technology) [113] were widely adopted. Besides, a new type of cell was found in the list, dendritic cells [26], as one of the sentinel cells. Dendritic cells are the most important and primary antigen-presenting cells of asthma. They take up the antigen, process it, and present the processed antigen to T cells [114]. Therefore, dendritic cell-related studies may be one of the breakthroughs in the treatment of asthma.

## 5. Conclusion and Future Trends

The number of cell research studies of asthma indexed by MEDLINE has increased rapidly. According to the ranking list of frequent stems, scholars paid more attention to experimental research, especially mechanistic research, rather than clinical research. The immunocyte studies and structural cell research are the two main directions. Eosinophils, mast cells, and T cells are the hot spots of immunocyte studies, while epithelia and SMCs are the hot spots of structural cell research. The research trend is closely linked with the development of experimental technology, including animal models. Early studies featured basic research, but immunity research has dominated in the recent decade with the development of genetic technology.

Based on the stem rankings of three decades, future trends can be predicted in the following aspects: (1) The distinct definition of asthma phenotypes associated with genetic characteristics will provide benefits for basic studies and clinical therapy. For instance, personalized medicine treatment tailored to individual's asthma phenotypes identified through biomarkers [115]. (2) Immunity research involving signaling pathways, regulatory mechanisms, targets with specific biomarkers, and so on at the gene level will provide more evidence for the pathogenesis of asthma. Meanwhile, the discovery of asthma biomarkers will contribute to characterize the population and associate the disease with environmental and therapeutic effects [116], as well as predict prognosis [117]. (3) The study of new cells regulating allergy, inflammation, or remodeling of airways, such as dendritic cells, type 2 innate lymphoid cells [118], and regulatory T cells [119], will bring the potential to provide therapeutic benefits.

## Figures and Tables

**Table 1:** Top 50 stems of three decades.

Ranking	1st decade		2nd decade		3rd decade	
	Stem	Frequency	Stem	Frequency	Stem	Frequency
1	eosinophil	147	inflamm	545	inflamm	1153
2	activ	115	express	425	respons	552
3	respons	113	eosinophil	358	express	537
4	inflamm	102	receptor	351	t	526
5	t	90	activ	341	activ	525
6	express	84	respons	339	receptor	508
7	bronchoalveolar	80	t	326	mice	476
8	role	76	role	321	role	429
9	lavag	75	induc	298	epitheli	422
10	mast	72	mast	232	induc	410
11	lymphocyt	71	smooth	229	muscl	406
12	atop	67	muscl	228	smooth	401
13	cytokin	61	epitheli	228	inhibit	401
14	receptor	60	cytokin	211	immun	336
15	releas	59	product	207	mous	333
16	induc	58	inhibit	205	murin	330
17	inhibit	55	allergen	200	mast	328
18	allergen	54	atop	177	eosinophil	294
19	product	53	protein	171	remodel	290
20	epitheli	53	sputum	168	signal	257
21	inhal	50	gene	166	protein	250
22	adhes	49	murin	160	children	245
23	mediat	47	mice	158	cytokin	244
24	inflammatori	46	pulmonari	148	inflammatori	235
25	children	45	inflammatori	146	suppress	234
26	histamin	43	hyperrespons	144	hyperrespons	232
27	muscl	41	children	136	pulmonari	230
28	leukotrien	40	inhal	136	gene	226
29	antigen	40	th2	118	product	224
30	smooth	40	remodel	118	th2	215
31	fluid	38	inhibitor	118	modul	213
32	sodium	38	mechan	116	dendrit	210
33	ige	37	develop	116	develop	207
34	peripher	34	rat	115	allergen	196
35	macrophag	34	allergeninduc	114	attenu	195
36	pulmonari	33	immun	113	pathway	190
37	vitro	32	lymphocyt	110	differenti	190
38	mechan	32	infect	106	target	185
39	select	31	sensit	102	novel	183
40	protein	30	novel	102	infect	177
41	therapi	28	mediat	100	mediat	176
42	glucocorticoid	28	peripher	100	regulatori	174
43	bronchoconstrict	28	signal	100	type	164
44	guinea	28	leukotrien	98	sever	164
45	chang	28	allergi	97	rat	164
46	pig	28	growth	97	promot	161
47	tcell	27	chemokin	96	inhibitor	156
48	hyperrespons	27	modul	93	allergi	156
49	immun	27	mous	93	mechan	151
50	modul	27	kinas	93	potenti	150
